# Review of visual analytics methods for food safety risks

**DOI:** 10.1038/s41538-023-00226-x

**Published:** 2023-09-12

**Authors:** Yi Chen, Caixia Wu, Qinghui Zhang, Di Wu

**Affiliations:** 1 Beijing Key Laboratory of Big Data Technology for Food Safety, Beijing Technology and Business University, Beijing, 100048 China; 2 National Measurement Laboratory: Centre of Excellence in Agriculture and Food Integrity, Institute for Global Food Security, School of Biological Sciences, Queen’s University Belfast, Belfast, Northern Ireland UK

**Keywords:** Agriculture, Risk factors

## Abstract

With the availability of big data for food safety, more and more advanced data analysis methods are being applied to risk analysis and prewarning (RAPW). Visual analytics, which has emerged in recent years, integrates human and machine intelligence into the data analysis process in a visually interactive manner, helping researchers gain insights into large-scale data and providing new solutions for RAPW. This review presents the developments in visual analytics for food safety RAPW in the past decade. Firstly, the data sources, data characteristics, and analysis tasks in the food safety field are summarized. Then, data analysis methods for four types of analysis tasks: association analysis, risk assessment, risk prediction, and fraud identification, are reviewed. After that, the visualization and interaction techniques are reviewed for four types of characteristic data: multidimensional, hierarchical, associative, and spatial-temporal data. Finally, opportunities and challenges in this area are proposed, such as the visual analysis of multimodal food safety data, the application of artificial intelligence techniques in the visual analysis pipeline, etc.

## Introduction

Risk analysis and prewarning (RAPW) is an essential part of food safety regulation, which can improve the quality and cost-effectiveness of food safety regulation and identify food safety risks at early stages. Food safety risk analysis is a systematic approach for analyzing chemical, biological, and physical hazards in food, including risk assessment, risk management, and risk communication^1^. Food safety prewarning is used for the prediction of future food safety events or risks derived from historical data by analysis models^2^. In recent years, progress in data and computer sciences has successfully reshaped the traditional algorithms in data management and processing, and data-driven food safety risk analysis has developed into one of the key approaches for food safety risk identification and early warning. With advances in inspection technology and improvements in regulation methods, the size and diversity of food safety data have exploded^3^, posing emerging challenges to data analysis techniques.

Although deep learning (DL) has shown significant advantages in the automatic learning of data characteristics and patterns, there are still some knowledge gaps in the practical application of food safety. For one thing, human experience and knowledge have not been considered in most automated analysis methods^4^, while the process of food safety risk analysis and decision-making cannot be separated from the full online participation of domain experts. Both in determining the degree of contamination and safety risk of food and in issuing regulatory orders based on early warning results, which require rich domain knowledge and regulatory experience. For another thing, the current popular data analysis methods represented by machine learning (ML) and DL, due to their complex principles and difficult-to-interpret output results, make many regulators in food safety skeptical about their assessment and warning results, and still require further expert judgment and confirmation before they can be applied in practice. Visual analytics, which has emerged in recent years, uses visual interactive interfaces as a channel to integrate human and machine intelligence into the data analyzing process in a visual way^5^. It can help people explore, understand, and analyze large-scale data in speed and accuracy to accomplish analytical reasoning and decision-making^6–8^. Furthermore, visual analytics is a human-in-the-loop approach and analysts can interact with the visual data interface through rich interactive tools to understand the distribution of food safety risks and assist in making regulatory decisions. It provides new ideas for food safety data analysis and has gradually become an important tool for food safety regulations.

As visual analytics gradually shows great potential in food safety, it is necessary to explore the latest advances in visual analytics methods for food safety risk to motivate researchers to propose more excellent solutions for visual analytics in food safety. Many recent reviews have reported the application of artificial intelligence (AI) techniques in food safety, such as AI in food adulteration detection^9^, ML in foodborne disease surveillance^10–12^ and in food safety monitoring and prediction^2,13^, DL in food science and engineering^4^, and text mining techniques in food science and nutrition^14^. However, only a few reviews have systematically provided a compendium of data analysis and visualization methods applied to food safety RAPW in the past decade. This paper proposes a categorization of visual analytics techniques for food safety RAPW based on the visual analytics pipeline (Table 1). It emphasizes the application in food safety RAPW of these techniques involved in each stage of the visual analytics pipeline. Firstly, we highlight the data sources, data characteristics, and analysis tasks in food safety. Then, we summarize the visual analytics method for food safety risk in the past 10 years, including classical data analysis methods for the four types of analysis tasks and the major visualization methods for the four types of data characteristics. Finally, we also discuss the opportunities and challenges for visual analytics in food safety RAPW.

**Table 1:** Visual analytics techniques commonly used in food safety RAPW over the last decade.

Data and Analysis Tasks			
Food Safety Risks	Data Sources	Data Characteristics	Analysis Tasks
Microbial Contamination	Sensors	Multiple Dimensions	Association Analysis
Pesticide and Veterinary Drug Residues	Online Databases	Hierarchical Structure	Risk Assessment
Heavy Metal Contamination	Satellite Imagery	Associated Relations	Risk Prediction
Illegal Additive and Fraud	Social Media	Spatial-temporal Distribution	Food Fraud Identification

Data Analysis Methods			
Association Analysis	Risk Assessment	Risk Prediction	Food Fraud Identification
Correlation Analysis	Qualitative Method	Traditional Machine Learning	Bayesian Network
Regression Analysis	Quantitative Method	Shallow Neural Network	Extreme Learning Machine
Association Rule Mining	Comprehensive Method	Deep Neural Network	Convolutional Neural Network

Visualization Methods			
Multidimensional Data	Associated and Hierarchical Data	Spatial-temporal Data	Visualization Interaction
Scatterplot	Node-linked Graph / Tree	Map-based Methods	Select / Filter / Navigate
Scatterplot Matrix	Adjacency Matrix	Timeline Methods	Overview + Detail
Parallel Coordinates	Space-filling Methods	Spatial-temporal Correlation	Focus + Context

## Survey method based on the visual analytics pipeline

This section details the visual analytics pipeline and the categorization of visual analytics techniques for food safety risk proposed in this review.

### Visual analytics pipeline

**Fig. 1:**
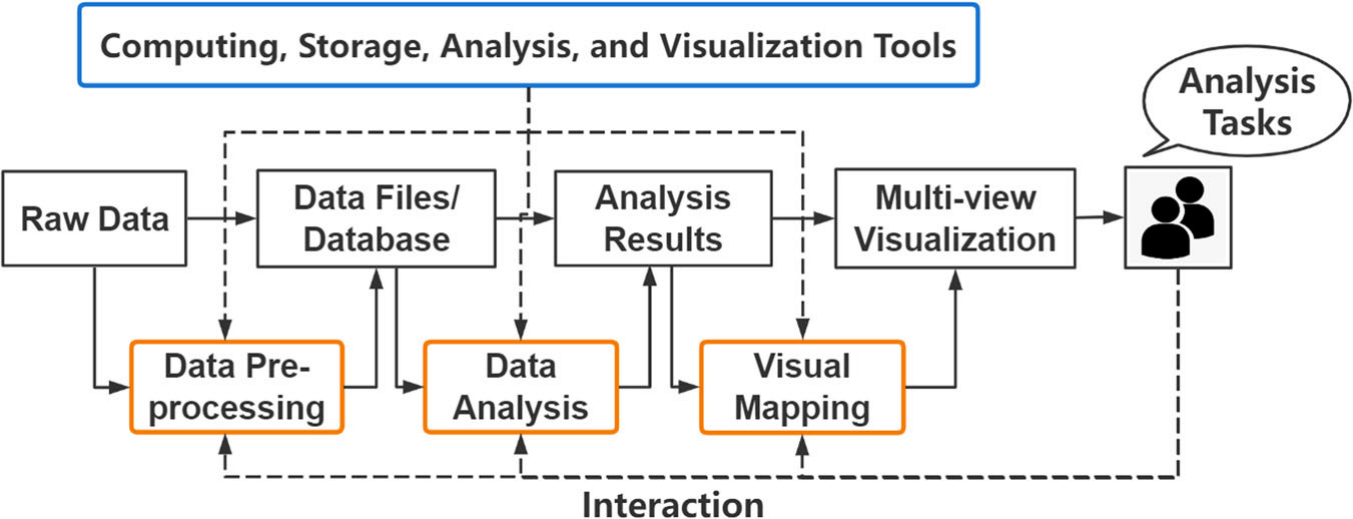
The complete visual analytics pipeline. It can be seen as a process in which data is transformed by a series of processing modules (brown boxes) that can be realized by a series of software tools (blue boxes). Specifically, the raw data is first stored in data files or databases after data pre-processing, then the analysis results are generated by data analysis methods, and finally multiple views are formed through visual mapping and presented to users. Users can interact with the data at various stages of this pipeline according to their analysis tasks to achieve a comprehensive analysis of the data.

Visual analytics, defined as a science of analytical reasoning facilitated by interactive visual interfaces^15^, is a new approach that integrates visualization, human intelligence, and data analysis. It maps complex data into easy-to-perceive graphs, symbols, colors, textures, and other representations and provides interactive means to help people derive insight from massive, dynamic, ambiguous, and often conflicting data^6^. A complete visual analytics pipeline is illustrated in Fig. 1. Specifically, the data are first stored in data files or databases after pre-processing. Then, the in-depth analysis and exploration of the data through data analysis methods resulted in much valuable information, namely analysis results. Finally, multiple views are designed for intuitive presentation and interactive analysis of data and analysis results through visual mapping. Under their analysis tasks, users interact with the data at various stages of this pipeline, e.g. selecting and filtering the data to be analyzed and switching between different views to get insight into the data. Relevant computing, storage, analysis, and visualization tools are accessible at every stage of the pipeline. Visual analytics is a process of human-in-the-loop that integrates human and machine intelligence into the data analysis process in a visually interactive manner, acquiring the complementary advantages and mutual promotion of the two to support analytical reasoning and decision-making.

### Survey method and categorization

In this work, we focus on visual analytics techniques for food safety RAPW. Following the visual analytics pipeline, a systematic and comprehensive review of studies in this area over the last decade is performed, by covering more than 100 papers, in terms of food safety data and analysis tasks, data analysis methods, and visualization methods. The specific categorization is provided in Table 1.

#### Data and analysis tasks

The abstraction of data and analysis tasks is typically the first key step in visual analytics. In contrast with other data, food safety data involves rich domain knowledge, a wide range of data sources, and distinctive data characteristics and analysis tasks. Accordingly, we summarize the food safety risks, data sources, data characteristics, and common analysis tasks in food safety.

#### Data analysis methods

For different types of analysis tasks, various data analysis methods are usually required for RAPW. We categorize the data analysis methods applied to food safety RAPW by analysis tasks, including techniques in four aspects: association analysis, risk assessment, risk prediction, and food fraud identification.

#### Visualization methods

The key to visualization is the mapping from data into visualization elements. In selecting a suitable visualization mapping method, data characteristics, such as the type and semantics, should be considered. Therefore, we divide visualization methods by data characteristics, including multidimensional data visualization, associated and hierarchical data visualization, spatial-temporal data visualization, and interactive techniques in visualization.

The main discussion in this paper is around visual analytics methods for structured food safety data, while analysis methods for semi-structured and unstructured data are not covered.

## Data and analysis task in food safety

Data is the object of visual analytics and analysis tasks are the goal of visual analytics. Understanding the data sources, data characteristics, and analysis tasks in the food safety domain can help analysts select the appropriate data analysis methods and visualization techniques to support visual analysis of food safety risks. Therefore, this section provides a detailed description of data sources, data characteristics, and analysis tasks in food safety.

### Data sources

The health risks derived from food products can be direct consequences of exposures to biological and chemical hazards from the environment and human activities throughout the food production, delivery, and consumption chain. Food contamination can cause a wide range of illnesses from diarrhea to cancers, which will affect human health and even threaten human life. The global food safety and supply chain systems, from field to fork, have been challenged by emerging risks raised from microbial contamination, pesticide and veterinary drug residues, heavy metal contamination, illegal addition, and fraud^16^. This can directly lead to serious food safety incidents and scandals resulting in huge impacts against public health, the economy, and consumer confidences^17^. The complex nature of food safety issues has urged the modernization of a highly efficient monitoring system to cover the whole supply chain against potential hazards. Therefore, government departments worldwide have strengthened food safety monitoring and control along the whole supply chain from farm to table, which, in turn, has generated massive food safety data.

The main channels for obtaining food safety data are sensors, online databases, satellite imagery, and social media. Many smart devices and sensors have been applied in food safety data collection, such as chromatograph-mass spectrometers for pesticide residue detection, radio frequency identification (RFID) sensors for food safety quality traceability, and mobile devices for rapid food detection. Online databases contain numerous information related to food safety, such as risk information from monitoring procedures and alert systems, exposure information from consumer databases, and surveillance reports on animal and plant diseases. These databases are published by some authorities e.g., the World Health Organization (WHO), the United States Food and Drug Administration (USFDA), the European Food Safety Authority (EFSA)^18^, and the State Administration for Market Regulation (SAMR) of China. Satellite image data, such as ground and meteorological data collected by remote sensing satellites and drones in real-time, can be used to monitor food contamination and quality in different areas. Social media has received massive public attention, and can also be regarded as a potential source of food safety data. On these platforms (e.g., Weibo, TikTok, and Twitter), information, sentiments, and news reports related to food safety can spread globally among individuals within a few seconds^19^. Food safety data can also appear in different formats, including numbers, texts, and images which constantly grows in both quantity and explosive scale in the informatic era.

### Data characteristics

From the perspective of data sources, food safety data have begun to show the ‘5 V’ characteristic of big data^20^. The first V is volume. Food safety data are obtained from a wide range of sources, and the scale of data becomes tremendous over time, creating a huge challenge for data collection and storage. The second V is variety. The types of food safety data are diverse, covering numbers, text, and images, with structured, semistructured, and unstructured data. The third V is velocity. Food safety data are generated and processed quickly in real-time. The fourth V is veracity. Food safety data are inevitably subject to errors and uncertainties in the entire process ranging from collection to use. The fifth V is value. The value density of food safety data is relatively low, that is, minimal valuable information is submerged into massive invalid information, posing a considerable challenge for mining and analyzing valuable information.

From the perspective of data analysis, food safety data are multidimensional, spatial-temporal, hierarchical, and associative. Multidimensional indicates that food safety data have multiple attributes. For example, food safety sampling data contain multidimensional attributes such as the name of the food, the category it belongs to, the time of sampling, the manufacturer, the items inspected, and qualification or failure. Spatial-temporal refers to the spatial and temporal attributes of food safety data. Statistics on the spatial and temporal distributions of food safety data are essential for RAPW. Hierarchical refers to the fact that some food safety data are organized in a tree structure. For example, the food classifications, hazard classifications, and administrative divisions of a region all exhibit hierarchical characteristics. Associative means that the different attributes of food safety data may be related to each other in some way. For example, there are explicit or implicit associations between hazards and foods, hazards and regions, and foods and regions in food safety sampling data.

### Analysis tasks

With the necessity of food safety regulation, analysis tasks in food safety are summarized as follows.

#### Association analysis

With the numerous dimensions and complex relationships in food safety data, it is important to explore potential associations within the data through correlation analysis, regression analysis, and association rule mining to identify food safety risks and provide prewarning.

#### Risk assessment

Risk assessment is an important aspect of food safety risk analysis. Based on available monitoring results, establishing risk assessment models to scientifically evaluate food safety risks is one of the analysis tasks in food safety.

#### Risk prediction

The primary purpose of food safety regulation is to avoid food safety incidents as much as possible. Food safety risk prediction and trend analysis can help regulators to detect safety risks in advance at an early stage, identify risk factors, and dispose of them in time to avoid bringing harm to human life and health.

#### Food fraud identification

Food fraud identification is an essential food safety risk analysis method, which helps risk managers make relevant decisions to effectively reduce the risk of fraud in the food supply chain by identifying key factors that may lead to food fraud events and predicting the types of food fraud notifications.

## Data analysis methods in food safety

In the human-in-the-loop visual analytics pipeline, data analysis is the previous step in visual mapping. Data analysis refers to the statistical modeling of data to explore data characteristics and patterns based on the analysis task. In this section, we categorize common data analysis methods in food safety RAPW by the analysis task, as presented in Table 2.

**Table 2:** Data analysis techniques commonly used in food safety RAPW over the last decade.

Analysis Tasks	Analysis Techniques	Application Cases in Food Safety
Association Analysis	Correlation Analysis	Measure the correlation between soil properties and heavy metal fractionation^22^
	Regression Analysis	Predict the presence of contaminants in chicken meat^24^
	Association Rule Mining	Mine multi-drug resistance patterns^27^ and build prewarning models^28,29^
Risk Assessment	Qualitative Method	Evaluate pesticide residue contamination^31^ and food safety risks in fresh produce and salmon farms^32^
	Quantitative Method	Evaluate the risk of heavy metals^33,35^ and food quality risks^36,37^
	Comprehensive Method	Evaluate the level of pesticide residue contamination^38^, food safety risks^39^, and heavy metal contamination^40^
Risk Prediction	Traditional Machine Learning	Predict food risks^41–43,47–50^ and foodborne disease pathogens^44^
	Shallow Neural Network	Predict the leaching rate of heavy metals^51^, coliform amount^53^, and food risks^54–56,58,59^
	Deep Neural Network	Predict the risk of sterilized milk^60,62^, gastrointesti-nal incidence^61^ and cooked meat products^63^
Food Fraud Identification	Bayesian Network	Analyze important factors that affect food fraud events^64^ and predict the types of food fraud notifications^65,66^
	Extreme Learning Machine	Identify adulterated edible animal blood food^67^
	Convolutional Neural Network	Classify natural and artificially ripened bananas^68^ and turmeric powder images^69^

### Techniques for association analysis

Association analysis is an important analysis task in food safety. Through association analysis of food safety data, domain experts can grasp the implicit associations between attributes such as food and hazards and identify the main factors affecting food safety risks to support food safety risk identification, risk assessment, and risk prewarning.

#### Correlation analysis

In statistics, correlation measures the strength and direction of a linear relationship between two variables, which is often measured with a correlation coefficient, such as Pearson correlation coefficient and Spearman correlation coefficient^21^. The Pearson correlation coefficient is often used to evaluate the linear relationship between two continuous variables, while the Spearman correlation coefficient is used to evaluate the monotonic relationship between two continuous or ordinal variables. For instance, Zhang et al.^22^ used Spearman’s correlation analysis to assess the relationship between soil properties and heavy metal fractionation, and the correlation between metal concentrations in soil and in plants.

#### Regression analysis

Regression analysis is an analysis method that uses the statistical principles of data to mathematically process a large amount of statistical data and determine the correlation between the dependent variable and certain independent variables, establishes a regression equation (function expression) with good correlation, and extrapolates it to predict future changes in the dependent variable^23^. According to the number of dependent and independent variables, regression analysis can be classified into two categories: univariate regression analysis and multiple regression analysis. According to the functional expressions of the dependent and independent variables, regression analysis can be classified into linear and nonlinear regression. It is also applied in food science widely. For example, Wu et al.^24^ developed a successive projection algorithm (SPA)–multiple linear regression (MLR) classifier based on optimal performance thresholds for the automatic prediction of the presence of contaminants in chicken meat, achieving a 100% true positive rate (TPR) and 0.392% false positive rate (FPR).

#### Association rule mining

Association rule mining, in data mining, is a popular method for discovering interesting relations between variables in large datasets. It can explore the relationships between variables from a large amount of food safety data. The Apriori algorithm^25^ is a typical association rule mining algorithm for breadth-first search^26^ and it uses prior knowledge to mine association rules between data. Cazer et al.^27^ applied the Apriori algorithm to mine multi-drug resistance patterns of chicken-derived *Escherichia coli* in antibiotic susceptibility experiment data. The Apriori algorithm was applied by Wang et al.^28^ to mine the temporal order and causal relationships between real-time monitoring data of the food supply chain. To help quality managers make more scientific food safety decisions, Jacobsen et al.^29^ developed a monitoring system that integrates transferable association rule models and data visualization techniques. This shows that the combination of visualization techniques and data analysis methods can provide an in-depth analysis of data mining results to support more effective decision-making.

### Techniques for risk assessment

Risk assessment is an important foundation for food safety regulation, and it can provide references for food safety decision-making. It refers to the risk analysis and ranking of biological, chemical, and physical hazards in food and food-related products^30^. Risk assessment methods are divided into qualitative, quantitative, and comprehensive assessment methods.

#### Qualitative assessment

Qualitative risk assessment analyzes and determines risks based on the experience and knowledge of experts. The expert assessment method (EAM) and the Delphi method are typical approaches. The EAM is an early and widely used assessment method that relies on the scoring of several experts based on their experience and expertise to make an assessment, such as using the EAM to set weights for each attribute in pesticide residue contamination assessment^31^. Soon et al.^32^ applied the Delphi method to assess food safety risks in fresh produce and salmon farms in the UK.

#### Quantitative assessment

Quantitative risk assessment refers to calculating the quantitative values of risk indicators to describe the risk level using data models, including the entropy method (EM), the Nemerow pollution index (NPI), and fuzzy comprehensive evaluation (FCE).

EM is usually combined with the comprehensive assessment method to perform risk assessment in food safety, and the related work is presented in the Section *Comprehensive assessment*. NPI is a weighted multi-factor pollution index, which emphasizes the most polluting factors while also considering the contribution of other factors in the assessment system^33^. This method first calculates the single-factor pollution index, then computes the comprehensive pollution index using the maximum and the average of the single-factor pollution index, such as calculating the heavy metal contamination risk of edible mushrooms^34^ and vegetables^35^. Yet, the comprehensive index calculated by the NPI method is susceptible to distortion due to the influence of the maximum value. Hence, FCE is mostly used for comprehensive assessment at present. FCE converts qualitative assessment into quantitative assessment by the affiliation theory of fuzzy mathematics; that is, it uses fuzzy mathematics to perform an overall assessment of objects that are subject to multiple factors. It can better solve fuzzy and difficult-to-quantify problems and is mostly combined with other quantitative methods for food safety risk assessment. Tanima and Madhusweta^36^ developed a mathematical model based on FCE and criticality analysis, called failure mode effects and criticality analysis (FMECA), for evaluating quality risk levels in the food supply chain. Wei et al.^37^ used the fuzzy analytic hierarchy process based on an optimal consistency matrix to evaluate the risk of milk knot samples qualitatively and quantitatively.

#### Comprehensive assessment

Qualitative or quantitative methods alone are insufficient for achieving accurate risk assessment, and thus, comprehensive assessment methods that combine qualitative and quantitative methods, such as analytic hierarchy process (AHP) and the best worst method (BWM), are more widely used in risk assessment.

AHP combines the experience of domain experts and mathematical models to determine the weight of each risk indicator. It can determine the indicator weights more effectively when there are many risk indicators for complex domain problems. However, since each risk indicator’s weight depends on its importance given by the experts, this makes its assessment results highly subjective. BWM reduces the involvement of domain experts by determining the best and worst indicators, making its results more objective. These two methods are frequently combined with EM for a comprehensive assessment of food safety risks, such as the AHP-E for the comprehensive assessment of pesticide residue contamination^38^, a risk assessment method combining entropy-weighted AHP and quality control analysis methods^39^, and a comprehensive assessment model that combines BWM and EM for quantitatively evaluating heavy metal contamination^40^.

### Techniques for risk prediction

The frequent occurrence of food safety incidents has brought considerable attention to the study of food safety risk prediction. Risk prediction typically refers to building suitable prewarning models to predict the risk value or risk level of newly detected food samples. The prediction results are crucial for identifying high-risk foods and hazards in advance and taking measures to curb the food safety risk.

#### Traditional machine learning

ML learns experiences from data and builds models to make predictions or decisions. Much research has demonstrated that ML algorithms are effective tools for food safety risk prediction^3,18^. The traditional ML algorithms are the classic machine learning methods before the emergence of DL. Support vector machine (SVM), hidden Markov model (HMM), gradient boost decision tree (GBDT), light gradient boosting machine (LightGBM), and Bayesian network (BN) were commonly used traditional ML algorithms in food safety.

SVM converts a prediction problem into a convex optimization problem to generate construct prediction rules in high-dimensional space for risk-level identification. Ma et al.^41^ developed a method base on parallel SVM for dairy production risk prediction and achieve 90% prediction accuracy. HMM is an important probabilistic model for serial data processing and statistical learning and has also been applied to food safety risk prediction. Han et al.^42^ proposed an HMM model based on gray relation analysis (GRA) for the risk prediction of sterilized milk. To take full advantage of the data features of large quantity and high dimension, Gao et al.^43^ applied LightGBM to predict the food risk by weighting and normalizing the feature values of discrete and continuous attributes. By comparing multiple ML methods, Wang et al.^44^ found that the GBDT model achieved the highest accuracy in identifying four pathogens: *Salmonella*, Norovirus, *E. coli*, and *Vibrio parahaemolyticus*.

BN is a probabilistic graphical model that combines Bayesian statistics, decision theory, and graph theory^45,46^. It is appropriate for analyzing uncertain probabilistic events and making decisions that conditionally rely on multiple control factors. BN has been widely used in food safety risk prediction, such as predicting the levels of microbial contamination and toxic and hazardous substances in food products^47^; predicting food safety hazards in herbal and spice products imported to the Netherlands^48^; and analyzing the influences of agricultural, climatic, and economic factors on food safety hazards in fruits, vegetables, and dairy products^49,50^.

These traditional ML algorithms offer clear algorithmic inference and prediction processes that have quick learning curves and don’t require large amounts of training data. However, the performance of most traditional ML algorithms is limited by the accuracy of manually extracted features. While DL algorithms attempt to obtain high-level features directly from the data, which can significantly improve the performance of risk prediction.

#### Shallow neural network (SNN)

Another popular technique for building prediction models is the artificial neural network (ANN)^51^, which is appropriate for handling food safety data with ambiguous correlations between various variables. Early ANN, also known as SNN, was a shallow model with only one hidden layer. Backpropagation (BP) neural networks, radial basis function (RBF), and extreme learning machines (ELM) are frequently employed SNNs in food safety.

A BP neural network is a multilayer feedforward network with error backward transmission. It excels at addressing nonlinear and uncertainty problems because of its powerful nonlinear mapping capability. Many researchers have applied BP neural networks to predict food safety risks, including predicting the leaching rate of heavy metals in tea^52^, determining coliform amounts in food^53^, judging whether a sample is qualified^54^, assessing dairy quality and safety risk^55^, and establishing a contamination index for six categories of hazards in food, containing compounds, pesticide residues, veterinary residues, heavy metals, microorganisms, and pathogenic bacteria^56^. Yet BP neural networks are slow in training and prone to the local minimum due to the use of gradient descent to obtain the minimum error. RBF neural networks^57^ proposed by Broomhead and Lowe have faster training speeds and can avoid getting trapped in the local minimum. Geng et al.^58^ proposed a food safety risk warning method based on agglomerative hierarchical clustering (AHC) and RBF neural networks for the risk prediction of meat products. ELM is faster to compute and more generalizable than traditional neural networks since it does not require parameter tuning during training and directly solves output weights without iterations. A food safety risk prediction model integrating AHP and ELM was applied to predict the safety risk of dairy products in one province of China in 2012 and 2014^59^. This model with multiple inputs and multiple outputs can simultaneously predict three types of hazard risks, namely pathogenic bacteria, heavy metals, and chemical contaminants, in food products. However, the application of SNNs in food safety is limited by the ability to express complex functions and the generalization ability for complex problems.

#### Deep neural network (DNN)

DNNs can approximate complex nonlinear functions and make more accurate predictions for future events via a deep network with many hidden layers. Some researchers have applied DNNs to predict food safety risks, including deep RBF (DRBF), deep denoising autoencoder (DDAE), and long short-term memory (LSTM) networks.

Geng et al.^60^ proposed a prewarning method combined with AHP and DRBF to predict and analyze the risk of sterilized milk sampled from a Chinese province in September 2014. It has been shown that their approach outperforms BP in terms of learning performance and generalization accuracy. A DDAE model based on EBO (ecogeography-based optimization) was designed by Song et al.^61^ to predict the incidence of gastrointestinal diseases caused by food contamination. Their experimental findings demonstrated that their evolutionary deep learning model outperformed the shallow ANN model and DDAE with other learning algorithms on a real-world dataset. LSTM neural networks can learn the dependencies among long sequences to make accurate predictions. A risk warning model that combined an LSTM neural network and sum product-based AHP (AHP-SP) was tested on sterilized milk data from a food testing facility in China by Geng et al.^62^. Compared with BP and RBF neural networks, their technique demonstrated superior accuracy in predicting trends in food safety risk. Chen et al.^63^ proposed a risk prediction method for food safety called TabNet-GRA, which combines a specialized deep learning architecture for tabular data (TabNet) with a grey relational analysis (GRA) to predict food safety risk. Their comparative evaluation unequivocally demonstrated the superiority of the TabNet-based prediction model over six typical models (RF, GBDT, XGBoost, BP, ELM, and RBF) under equivalent conditions.

### Techniques for food fraud identification

Many ML and DL methods are also widely used for food fraud identification, including BN, ELM, and Convolutional Neural Network (CNN). BN models have been applied to identify the crucial factors that may contribute to the occurrence of food fraud events^64^ and predict the types of food fraud notifications^65,66^. These findings can help risk managers identify the principal factors that influence food fraud, and thus, enhance their ability to risk management and food fraud risk mitigation. ELM and newly introduced CNNs are currently commonly used supervised learning algorithms in food fraud classification, such as the extreme learning machine regression (ELMR) model for identifying adulterated edible animal blood food (EABF)^67^, the CNN for classifying natural and artificially ripened bananas^68^ and the improved CNN for classification of turmeric powder images to detect fraud^69^. These studies suggest that DL has emerged as an effective method for assessing food quality and identifying fraud.

### Data analysis tools

Conventional data analysis tools include Excel, Statistical Analysis System (SAS)^70^, Statistical Product and Service Solutions (SPSS)^71^, R^72^, MATLAB^73^, and Python^74^. Among them, non- programmatic data analysis tools, including Excel, SAS, and SPSS, support fundamental statistical analysis functions and have user-friendly graphical interfaces that are more suitable for novices. However, these three analysis tools are less capable and efficient in processing data, and the data analysis models provided are not flexible enough to re-tune the models as necessary. Programmatic tools, including R, MATLAB, and Python, provide a variety of functions for mathematical calculations and statistical analysis, enabling users to perform data analysis flexibly and even create new statistical methods that meet their needs. The drawback of programmatic tools is that they require some programming skills of users. Therefore, in practice, analysts should choose the appropriate data analysis tool according to their knowledge background and the needs of the practical application.

Although data analysis methods represented by ML have provided many effective algorithms and models for food safety RAPW, there are still some knowledge gaps in the practical application of food safety. Many experts in the food safety field find it challenging to understand the distribution characteristics of the raw data and to judge the accuracy of the analysis results when making decisions because the principles and outputs of most models are difficult to explain in human-comprehensible language. Additionally, the data analysis process in food safety usually requires the full online participation of experts from analyzing raw data and adjusting model parameters to make effective decisions. However, the existing analysis methods are mostly fully automated processes, lacking the participation of expert knowledge and experience. To close the gap in data analysis, it is necessary to introduce some interpretable techniques (e.g. visualization) to make the data analysis process visual and engageable.

## Visualization methods in food safety

In the visual analytics pipeline, both raw data and data analysis results can be presented to the user through visualization techniques for further exploration and analysis. Visualization is defined as the communication of information by using graphical representations to help people accomplish analytical tasks more effectively^75^. In this section, we introduce visualization techniques and their applications in food safety RAPW based on data characteristics, as presented in Table 3.

**Table 3:** Visualization techniques commonly used in food safety RAPW over the last decade.

Categorization	Visualization Techniques	Application Cases in Food Safety
Multidimensional Data Visualization	Scatterplot	Show the correlations of two attributes in red wine data^78^
	Scatterplot Matrix	Analyze the correlations among multiple nutrients in food^79^
	Parallel Coordinates	Compare the differences between multiple maximum residue limit standards^82^ and rank agricultural products by multiple indicators^31^
Associated and Hierarchical Data Visualization	Node-linked Graph/Node-linked Tree	Show the association between food and hazards^83,84^ and present the classification of foods, food additives, and products^85^
	Adjacency Matrix	Represent the detection relationship^86^
	Space-filling Methods	Represent the distribution of pesticide residues^87^, the differences of multiple maximum residue limits^82,91^, and the association between hazards and products^88–90^
Spatial-temporal Data Visualization	Map-based Methods	Analyze the spatial distribution of pesticide residue^92,94^
	Timeline Methods	Capture temporal trends and seasonal patterns of salmonellosis^118^
	Spatial-temporal Correlation Methods	Capture the geographical distribution characteristics and the development trend of food attributes over time^87^
Visualization Interaction	Select / Filter / Navigate	Quickly locate highly contaminated agricultural products and pesticides^86^ and compare maximum residue limit standards from different countries or regions^82^
	Overview + Detail	
	Focus + Context	

### Multidimensional Data Visualization

Multidimensional data are widely available in food safety. However, the physical limitations of display devices and the human visual system do not allow direct display and rapid recognition of structures with dimensions of more than two. For these reasons, various visualization methods have been introduced to convey multidimensional structural information visually on a 2D screen, including scatterplots, scatterplot matrices, and parallel coordinates.

**Fig. 2:**
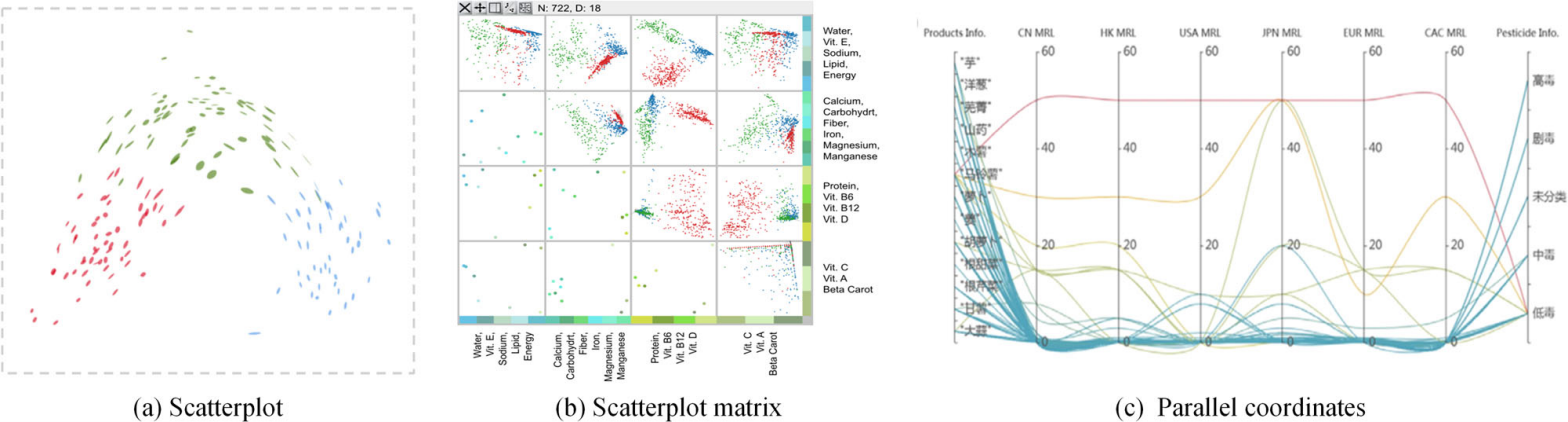
Examples of multidimensional data visualization. **a** Scatterplot shows the correlation between two attributes in red wine^78^. **b** Scatterplot matrix displays the correlation between multiple nutrients in food^79^. **c** Parallel coordinates compare MRL values of pesticides for various agricultural products in different countries or regions^82^.

The scatterplot represents data points on rectangular coordinates to illustrate the relationship between two variables. A scatterplot matrix^76,77^ is an extension of the scatterplot; it is used to reveal the relationship between two of all the dimensions in the data. A scatterplot matrix for *n*-dimensional data consists of several scatterplots arranged in an *n × n* matrix following a certain order, wherein each scatterplot is drawn from every two dimensions of the *n* dimensions. Multidimensional visualization will lead to visual clutter due to too many dimensions, and it typically requires combining dimensionality reduction methods for visualization, such as PCA, multiple dimensional scaling (MDS), and self-organizing maps (SOMs). Bian et al.^78^ proposed a multidimensional projection method based on implicit function differentiation, projecting the basis vectors obtained via PCA from the wine dataset onto a 2D plane to form a scatterplot, as shown in Fig. 2a. To explore the data correlation and dimensionality correlation of high-dimensional data, Yuan et al.^79^ proposed a dimensional projection matrix and applied it to analyze food nutrition datasets, wherein each dimension represents a nutrient, as shown in Fig. 2b. The United States Department of Agriculture (USDA) food nutrition dataset is first projected onto a 2D scatterplot via MDS. Then, the dimensions are divided into four mutually exclusive groups on the basis of the clustering characteristics in this plot. Finally, a dimensional projection matrix is formed to help users explore and analyze correlations among multiple nutrients in food.

Parallel coordinates represent data attributes through a set of parallel axes, while a data object is represented by a line through each axis wherein parallel axes are vertical or horizontal lines distributed at equal distances^80,81^. The use of parallel coordinates to present multi-attribute data can help users analyze the correlations between dimensions and the distribution characteristics in data. Chen et al.^82^ used parallel coordinates to show the maximum residue limit (MRL) values of agricultural products in six countries (or regional organizations), helping users compare the differences among multiple MRL standards. However, as the number of data dimensions and data objects increases, visual clutter will occur in traditional parallel coordinates. To overcome the drawback in which parallel coordinates with mass data experience difficulty in quickly obtaining attribute values, Chen et al.^31^ designed a visualization method that combined parallel coordinates with a bar chart. This method supports the ranking of several agricultural products by using multiple pesticide residue assessment indicators. They also developed a visual analytics system with multi-attribute ranking, enabling users to comprehensively explore the ranking of various agricultural products contaminated with multiple pesticides through interactive means, such as multi-view coordination, data filtering, and attribute selection.

### Associated and hierarchical data visualization

Food safety data exhibit typical associated and hierarchical characteristics. Hierarchy is a specific form of association that is primarily expressed as inclusion and subordination relationships among entities. Visualization methods for association and hierarchical data are similar, and thus, we discuss both in this subsection. Three types of methods are available for visualizing associated and hierarchical data: node-linked method, adjacency matrix, and space-filling method.

#### Node-linked methods

A node-linked method is a typical visualization method for associated and hierarchical data. It uses nodes of different shapes to represent entities (content information) and the lines between nodes to represent the relationship among entities (structural information). Graphs can be used to describe the association relationship among entities, while trees are used to describe the hierarchical relationship among them. The node-linked method can clearly show the associated hierarchy of small-scale data but with low space utilization in general. Therefore, interactive techniques are required for presenting large-scale associated and hierarchical data.

To analyze the contamination of various food by hazards and their geographical distribution, Gao et al.^83^ used a network graph to show the association of food, region, and hazards. Similarly, Yang et al.^84^ used the node-linked method to associate a food product with its detected nonconforming hazards. The higher the detection frequency of hazards, the thicker the connecting link in this graph. This method can help regulators locate key regulatory foods and hazards. For hierarchical relationships, Qi et al.^85^ used node-linked trees to construct a food classification map that presents the classification of food and food additives.

#### Adjacency matrix

The adjacency matrix is one of the visual representations of associated data. It is an *N* by *N* grid (where *N* is the number of nodes), where position (*i*, *j*) represents the weight of the link between nodes *i* and *j*. Chen et al.^86^ proposed an ordered matrix representation method for the visual analytics of associated data in Fig. 5. This method represents the detection relationship between agricultural products and pesticides in a matrix heatmap. In this heatmap, each row represents a pesticide, while each column represents an agricultural product. The color of cells is mapped onto the content of the pesticide residue. The darker the color, the higher the pesticide residue content. The rows and columns in matrix A are arranged alphabetically to facilitate users in searching by name. The rows and columns in matrix B are organized using the RW-Rank algorithm proposed in the paper. This algorithm assists users in quickly locating pesticides with high residue levels and heavily contaminated agricultural products.

#### Space-filling methods

A space-filling method is a visualization method for hierarchical data that uses chunked areas of various shapes to represent data. The hierarchical structure of data is represented by enclosing relationships. In particular, parent nodes enclose children and grandchildren nodes, indicating a parent-child relationship in a tree. Space-filling techniques can maximize the use of display space and are represented by treemaps, circle packing, and radial rings.

**Fig. 3:**
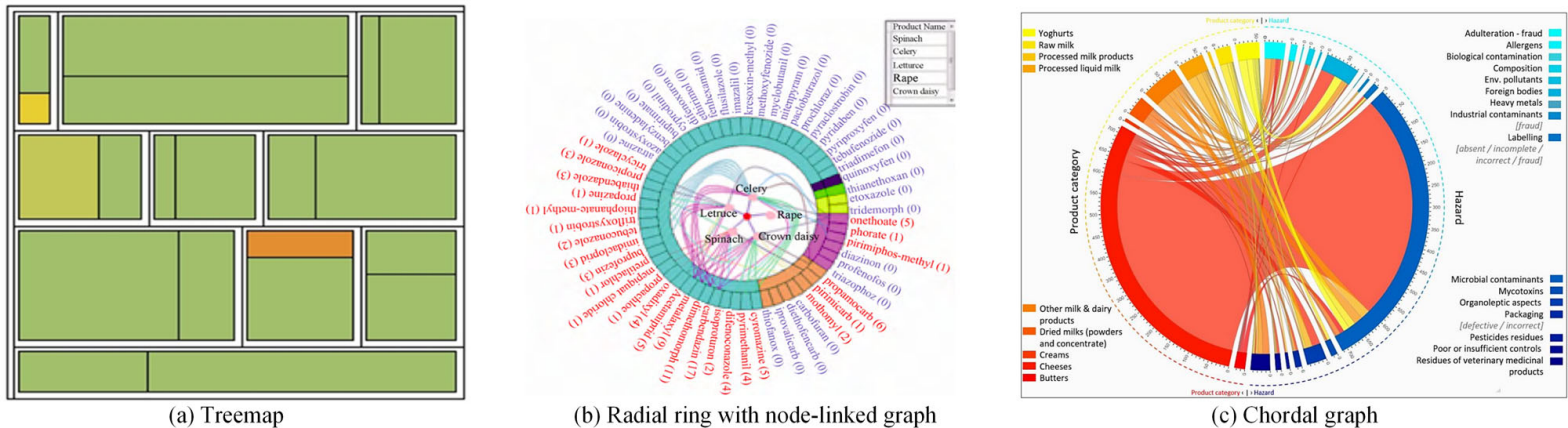
Examples of hierarchical and associated data visualization. **a** Treemap shows the distribution of pesticide residues detected in fruits and vegetables in 10 regions of Tianjin in January 2014^87^. **b** SONHC displays the hierarchy of pesticides with an external radial ring and the hierarchy of agricultural products with an internal node-linked tree^88^. **c** Chordal graph presents the association between hazards and dairy products^89^.

A treemap is a recursive partitioning in rectangular space. It consists of a series of nested rectangles with sizes that are proportional to the corresponding node attribute values. A large rectangle represents a branch of the data tree; it is subdivided into smaller rectangles to represent the size of each node within this branch. Chen et al.^87^ used a treemap to represent the distribution of pesticide residues detected in fruits and vegetables in 10 regions of Tianjin, China (Fig. 3a). Each large rectangle in the treemap represents a region of Tianjin, and one of the small rectangles in each large rectangle represents fruits while the other represents vegetables. The color indicates the rate of excess pesticide residues, i.e., a higher excess rate is closer to red. The treemap has been proven to meet the requirements for the analysis of hierarchical structures and associative relationships in the field of food safety. It maximizes the use of display space but is not as clear as a node-linked graph in representing hierarchical structures.

Circle packing combines the advantages of node-linked graphs and treemaps by using the area of the circle to represent the attribute values of the nodes and the nested relationship of the circle to represent the hierarchical relationship among the nodes, with all child circles contained in the parent circle. Chen et al.^82^ used two circle packings in juxtaposition (Fig. 6, view A2) to visualize two MRL trees, enabling users to compare and analyze the MRL standards of two countries or regions. MRL standards are classified by agricultural products (which is a tree structure), and the problem of comparing two MRL standards can be transformed into a problem of comparing two trees. A nested structure is used to describe the hierarchy of agricultural product classification, and the area of the circle is used to represent the number of MRL standard records for an agricultural product.

A radial ring is also a space-filling method, but its internal space utilization is low. Therefore, to fully utilize the space near the center of a radial ring, Chen et al.^88^ designed a combined method based on the radial ring and node-linked methods, called sunburst with ordered nodes based on hierarchical clustering (SONHC). The designed method can simultaneously display two types of hierarchical data, as shown in Fig. 3b. It uses the outer radial ring to display the hierarchical structure of pesticides, and the inner node-linked tree to display the hierarchical structure of agricultural products. It establishes the association between agricultural products and detected pesticides through the link to help relevant analysts examine the relationship between them. A chordal graph is also a combination of the radial ring and node-linked methods. Postolache et al.^89^ designed a chordal graph to show the association between hazards and dairy products in Fig. 3c. Du et al.^90^ proposed a transformation-based graph, called TransGraph, for analyzing relations in a dataset by combining donut and radial rings. This graph supports the analysis of the association between agricultural products and pesticides and the hierarchical association of pesticide residues. Luo et al.^91^ designed a sunburst with an embedded chordal graph (SECG) and an overlapping circular treemap (OCT) to lay the foundation for a comprehensive comparison of pesticide MRL standards.

### Spatial-temporal data visualization

The spatial-temporal analysis of food safety data with spatial and temporal characteristics enables researchers to identify the geographical distribution and trends over time of food safety risks. Visualization methods for spatial-temporal data include map-based methods, timeline methods, and spatial-temporal correlation methods.

**Fig. 4:**
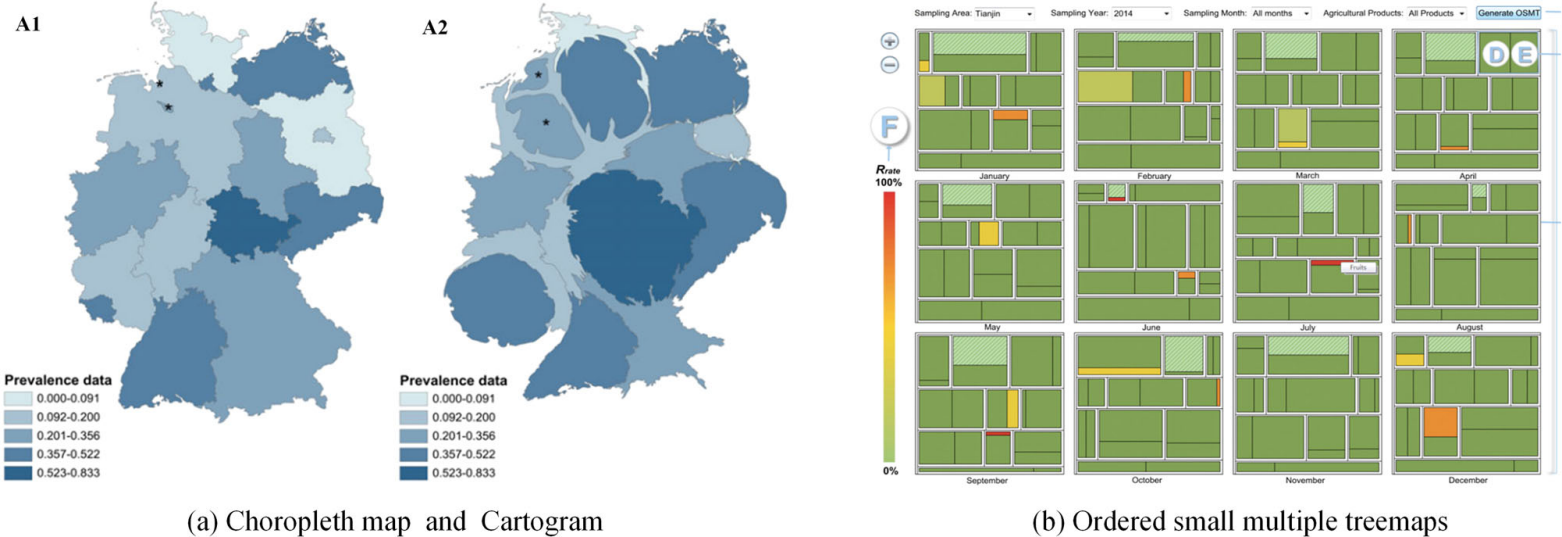
Examples of spatial-temporal data visualization. **a** Prevalence rates of *Campylobacter* spp. in raw chicken meat in German are compared by choropleth map (A1) and Cartogram (A2)^94^. **b** Ordered small multiple treemaps show the excess rate of pesticide residues for fruits and vegetables in 10 areas of Tianjin for 12 months in 2014^87^.

Most spatial data involved in food safety are geographic area data ranging from countries and provinces to as small as a block or a supermarket. Representative visualization methods based on maps are choropleth maps and cartograms. A choropleth map assumes that the attributes of the data are evenly distributed within an area. It uses colors to represent the inherent patterns of the data, such as an atlas of pesticide residue levels in fruits and vegetables on the market in China compiled by Pang et al.^92^. Cartograms have been proposed to solve the problem of asymmetry between data distribution and geographic area size in choropleth maps. A cartogram can replace the real area of a region with the size of an attribute value and then distort and deform the geographic region to reflect quantitative characteristics more intuitively^93^. Plaza-Rodrıguez et al.^94^ used a cartogram to analyze the spatial distribution of the detection rates of *Campylobacter* spp. in retail raw chicken meat in Germany (Fig. 4a). The size of the states in the cartogram map was modified by prevalence, such that small geographic areas with a high prevalence can also receive attention.

The timeline method for time-series data can more intuitively reflect the patterns and trends of data changes over time and also show the data details. Time-series data are typically expressed in the form of a timeline. Simpson et al.^118^ proposed multiple multi-panel plots to capture temporal trends and seasonal patterns of salmonellosis. A multi-panel plot includes a rotating histogram of monthly rate frequency (left panel) and a time series of monthly rates (right panel) for salmonellosis in the United States from 1996 to 2017. Overlaid annual time series plots of monthly rates, a box plot of average monthly rates for the 22-year period, and overlaid yearly radar plots of monthly rates comprise another multi-panel plot for displaying seasonal signatures of salmonellosis monthly rates in the United States from 1996 to 2017.

Spatial-temporal correlation is a common data analysis task in food safety. Exploring spatial-temporal correlations in data enable analysts to grasp the geographical distribution characteristics and the development trend of food attributes over time. Chen et al.^87^ proposed a visualization method, called ordered small multiple treemaps (OSMT), which used juxtaposed treemaps to visualize the changes in pesticide residue exceedance rates of fruits and vegetables in 10 regions of Tianjin for each month in 2014 (Fig. 4b). Twelve treemaps are juxtaposed to represent the changes in twelve months, and each treemap represents the exceedance rate of pesticide residues in fruits and vegetables in ten regions of Tianjin within a certain month.

### Interaction in visualization

Interaction, where user actions cause the view to change, is crucial for building a visualization system that handles complexity^6^. When datasets are large enough, the limitations of both people and displays preclude just showing everything at once, changing the view over time, and switching among multiple linked views through the interaction are good solutions. For example, an interactive visualization tool can support investigation at multiple levels of detail, ranging from a very high-level overview down through multiple levels of summarization to a fully detailed view of a small part of it.

A view that changes over time can dynamically respond to user input, rather than being limited to a static image. The common interaction operations in visualization are selection, navigation, and filtering. Selection, or marking data objects of interest, is commonly performed by selecting objects with interactive hardware, such as a mouse, keyboard, etc. Navigation, which typically involves the actions of zooming, panning, and rotating, refers to observing the different aspects of the dataset by changing the viewpoint. Filtering displays a part of the data based on certain conditions. Shneiderman proposed that the golden rule of visualization design is “overview first, zoom and filter, and details on demand”^95^. The basic idea is to first display an overview of the dataset and then show the details of interest to the user by zooming and filtering. The overview provides the user with a general impression of the data structure and other global information. The details are the information filtered through user interaction and help users to explore the data of their interest in depth. Consequently, the “overview + details” is one of the primary modes of visual interaction and makes delving deeper into the implied relationships between data become easier for the user.

**Fig. 5:**
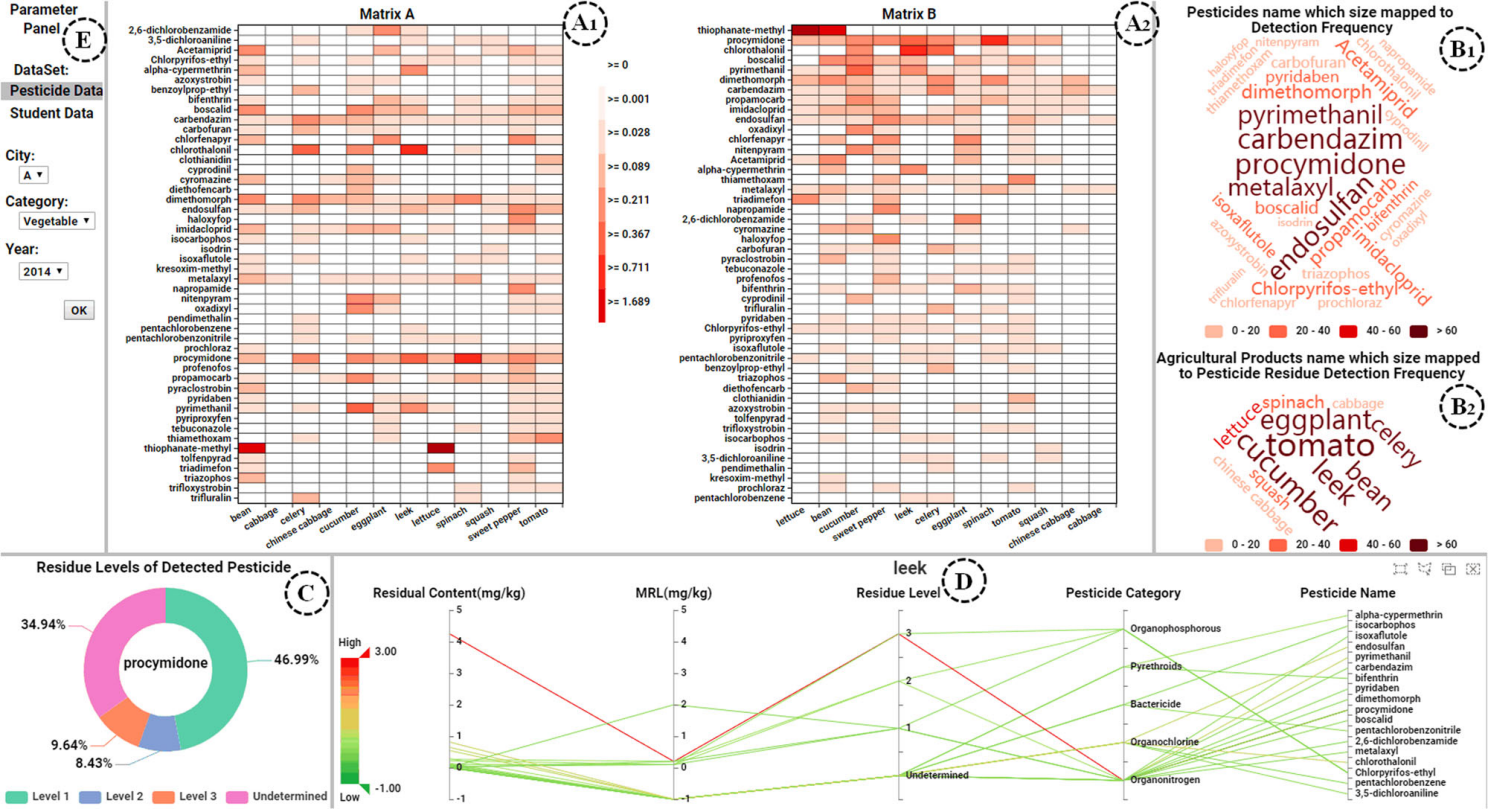
Interactive visual analytics system Rank-Vis for quickly locating pesticides with high residue levels and highly contaminated agricultural products^86^. After selecting the pesticide-residue dataset and filtering the data of interest in the parameter panel (View E), two matrix heatmaps for the product–pesticide association will be created in Views A_1_ and A_2_. View B_1_ and B_2_ are the word clouds, in which the sizes of the pesticide and agricultural product names are mapped to the pesticide residue detection frequencies. Pie chart in view C shows the percentages of four residue levels in different agricultural products selected by clicking their names in view B_1_. Parallel coordinate in View D shows the detailed information of a specific agricultural product selected by clicking its name in View B_2_.

Switching among multiple linked views is another popular choice in visual design. Multiple views juxtaposed side by side are spread out in space is an alternative to a changing view where the information is presented to the user. It mainly includes two methods: (1) the multiform design is to use a different encoding in each one to show the same data; (2) the small multiples coordination design involves partitioning the data between views. In practical analysis, users typically need to switch between multiple views and observe the dataset at different levels. Chen et al.^86^ proposed a Rank-Vis system with multi-view coordination to help users quickly locate highly contaminated agricultural products and pesticides with high residue levels, as shown in Fig. 5. Users can select a dataset and filter the data of interest in the parameter panel (View E). After selecting the data, two matrix heatmaps for the product–pesticide association will be created in Views A_1_ and A_2_. The word clouds on the right show the names and detection frequencies of pesticides (View B_1_) and agricultural products (View B_2_). When users click on pesticide in the word cloud (View B_1_), a pie chart is generated, showing the percentage of detected pesticides in four residue levels (View C). When users click on an agricultural product in the word cloud (View B_2_), a parallel coordinate is generated, presenting detailed information about the agricultural product (View D). Through interaction, users can quickly find agricultural products with serious contamination and pesticides with high residue levels, enabling them to analyze their categories, residue levels, and MRLs comprehensively.

**Fig. 6:**
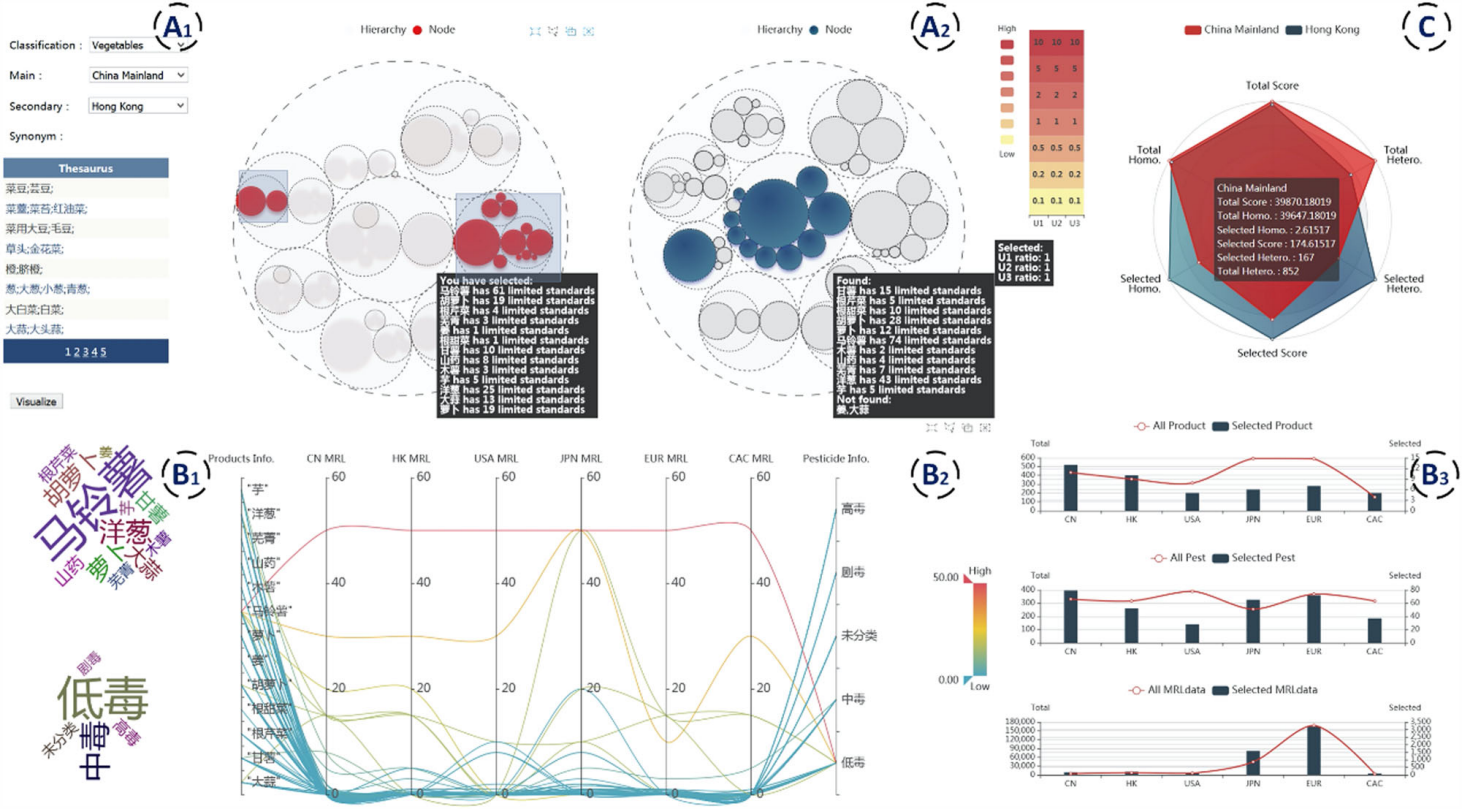
A Multi-comparable Visual Analytic system (McVA) for comparing the MRL standards of different countries or regions^82^. Users can select the product category and country or region in View A_1_, then compare the difference in the number of standard limits in two regions for each agricultural subcategory in View A_2_. The word cloud in View B_1_ and the parallel coordinates in View B_2_ show the MRL value of user-specified agricultural products and the distribution of pesticide toxicity. The bar chart in View B_3_ can helps users compare the quantities of agricultural products, pesticides, and standard limits in different regions. View C can help users compare the hierarchical structure of MRL standards in two regions from six dimensions.

Another multi-view coordination visual analytics system, called the multi-comparable visual analytics system (McVA), was proposed by Chen et al.^82^. It provides interactive techniques to help users compare MRL standards of two different countries or regions. As shown in Fig. 6, when users select the product category and country or region in View A_1_, View A_2_ will display the MRL standards for selected agricultural products in the selected regions by using two juxtaposed circle packings. Users can use the lasso tool to circle the subcategory of produce in View A_2_ and then compare the difference in the number of standard limits in two regions for each agricultural subcategory. The product data displayed in other views (View B_1_, B_2_, B_3_, C) are updated as the user circles the data. The radar in View C compares the hierarchical structure of MRL standards in two regions from six dimensions, including three indicators to assess the overall structure and three indicators to assess the substructures chosen by users. The word cloud in View B_1_ and the parallel coordinates in View B_2_ can effectively filter and show the MRL value of user-specified agricultural products and the distribution of pesticide toxicity. The bar chart in View B_3_ not only helps users compare the quantities of agricultural products, pesticides, and standard limits in different regions but also explore the relationship between the selected and total data. Through an efficient interactive approach, McVA provides users with considerable convenience in comparing and analyzing two different MRL standards in a comprehensive and multi-level manner.

### Visual Analytics Tools

Visual analytics tools can generally be divided into two categories: interactive and programmatic visualization tools. Interactive visualization tools typically complete the editing and drawing of visual charts by dragging data and selecting templates. Excel is the most common and basic visualization software. However, it can only draw simple visual graphs with fixed graph styles, such as bar charts and pie charts. Tableau^96^ is an easy-to-use visualization software that allows users to perform data analysis and create visual charts in a WYSIWYG manner, without programming. DataV^97,98^ is a mature drag-and-drop chart creation production built upon a web-based architecture. It allows users to edit and save their visual creations in an online editor without professional knowledge of programming. Programmatic visualization tools are frequently a JavaScript library that can be introduced into visual design projects to support the autonomous creation of interactive visual interfaces. Echarts^99^ is an open-source visualization library that is compatible with most current browsers; it can provide intuitive, interactive, and highly customizable data visualization charts. Its website^100^ also contains a rich set of examples and tutorials that are easy to use. D3 (Data-driven documents)^101,102^ provides efficient data-driven document object model (DOM) manipulation and supports custom charts and complex interaction designs, which is widely used in the academic community.

## Opportunities and challenges

Although visual analytics has been widely applied to solving food safety problems and has achieved promising results, several challenges remain. Here, we discuss the major challenges and potential horizon for the applications of visual analytics in food safety RAPW.

### Visual analytics of multimodal food safety data

With advances in detection instruments and information technology, food safety data is not only growing rapidly in quantity but also diversifying in data types, such as digital, text, image, audio, video, etc. Deep learning, with a large number of successful cases in image processing, speech recognition, text mining, object detection, and so on, has recently been the data analysis tool to solve the problems and challenges in food domain, including food recognition, calories estimation, quality detection of fruits, vegetables, meat and aquatic products, food supply chain management and detection of food contamination^4^. However, only a few multimodal food safety data were used in visual analytics of food safety risk. This is because most existing visual analysis methods for food safety data focus on small-scale, single-source, single-modality data. Joint analysis of data from different sources and modalities is required to obtain complementary information in practical applications. Food knowledge graphs, which emerged in recent years, can transform huge amounts of multidisciplinary and heterogeneous food data from different sources into entities and relations in the form of graphs, and can effectively express the semantic relations between entities. They have been applied in the food science and industry for food search and question answering, personalized dietary recommendation, food analysis and visualization, and food traceability^103,104^. Multimodal data fusion can automatically analyze and synthesize multimodal data to accomplish the required decision-making and assessment tasks, showing great potential in food risk identification and monitoring. Therefore, a key trend in this industry is to explore broad-spectrum visual analytics techniques that combine data fusion, knowledge graph, text mining, image recognition, and video processing in the visual analytics pipeline for analysis of multimodal food safety data, enabling more accurate food safety assessments and prewarning.

### Application of AI in the visual analytics pipeline

AI represented by ML and DL has begun to be applied to improve performance at each stage of the visual analytics pipeline. In the data analysis stage, AI can offer precise and effective solutions for data preprocessing, transformation, projection, and other steps, while providing scientific models for food safety risk assessment and prewarning. Compared with traditional data analysis methods, DL methods can better solve classification, prediction, and recommendation problems in food safety. For instance, DL models like CNN and LSTM have achieved promising results^62,105^ in food identification, risk assessment, risk prediction, and healthy recipe recommendation. In the future, advanced DL models like Recurrent Neural Network (RNN), Transformer, Generative Adversarial Network (GAN), and Graph Neural Network (GNN) will be introduced to further enhance the processing and analysis capabilities of food safety data. In the visualization stage, AI techniques such as neural networks, knowledge graphs, and reinforcement learning are applied to learn data characteristics and analysis tasks, enabling the automatic selection of visualization design schemes that better match the requirements of data and analysis tasks^106–108^. For example, it is possible to automatically select mapping methods (e.g., scatterplot, parallel coordinates, and node-linked graph), layout (e.g., orthogonal and radial), and color scheme using LSTM, RNN, Translating Embedding (TransE), Deep Q-Network (DQN) and other models^109–112^. These techniques of automatically creating visualization schemes will considerably lessen the workload of visualization design for experts in food safety, enabling them to analyze and predict food safety risks more efficiently. In particular, the popularity of ChatGPT has led to a keen awareness that Artificial Intelligence Generated Content (AIGC) will lead the way to the latest paradigm in research. In food safety RAPW, AI automated design and generation of visualization schemes will also become a hot research topic. In summary, another significant trend in this industry will be the deep integration of AI and visualization approaches to achieve more scientific and effective food safety risk analysis and prewarning.

### Building and sharing of multimodal food safety database

Future AI-based visual analytics of multimodal food safety data will heavily rely on large-scale data. More specifically, food safety association visual analysis requires cross modal, cross spatial-temporal, and cross domain data to design charts, while ML and DL require a large amount of fine-grained food safety data to train models. Currently, detailed information on food classification, physicochemical properties of contaminants, sampling information, limit standards, and warning reports is available in some online databases released by food safety authorities (as described in Section *Data Sources*). Some food nutrition databases (typically represented by MADiMa^113^ and Nutrition5k^114^) released by academic research teams provide food images, ingredient information, and nutrient content together with the corresponding annotations and labels. Although these data serve as the foundation for association visual analysis of food safety risks, they are still insufficient for visual risk analysis and prewarning, in terms of data type, data granularity, and data content, and still need to be constantly updated and supplemented. In addition, the ability of a person, research team, institution, or country to collect data is limited^4^. It is essential to establish data sharing mechanisms to maximize the sharing of multimodal food safety data. Meanwhile, more efforts should be made to enable the entire industry compliance with General Data Protection Regulation (GDPR) rules to protect data privacy while allowing data sharing due to the sensitive nature of food safety data. In short, the building, accumulation, and sharing of multimodal food safety databases will be a long-term issue to be addressed for AI-based visual analytics of multimodal food safety data.

### More user-friendly visual analytics systems

Data visualization and interaction are two important means by which visual analytics can help users gain insight into the data. Designing easy-to-understand data visualization representations and user-friendly interactions are two important aspects in the development of visual analytics systems. Typically, most people working in food safety data analysis, with extensive food domain expertise and experience, are not computer domain professionals and not necessarily familiar with computer expertise, especially visualization techniques. Many existing visual analytics systems provide relatively complex visual representations that require a certain level of visualization knowledge learning and training for non-computer professional users to become proficient in their use. This prevents experts in food from focusing on domain issues and limits the widespread use of visual analytics systems in food safety. Therefore, it is an urgent problem to design visualization schemes that are more easily understood by domain users, such as improvements and combinations of their familiar scatter, bar charts, pie charts, and node-link diagrams. In addition, most interactions used in existing visual analysis systems are limited to traditional keyboard and mouse interactions, which do not give full play to human perception and cognitive abilities. Applying advanced human-computer interaction techniques, such as eye tracking, gesture recognition, speech recognition, and natural language processing, to develop more convenient and easy-to-use interaction methods that enable users to communicate more naturally with computer systems^115–117^, is also one of the future research opportunities. In conclusion, the design of more user-friendly (i.e., easier to understand and use) visual analytics systems based on specific data characteristics and analysis tasks in food science and engineering is also one of the future efforts.

## Discussion

In this paper, we reviewed data analysis methods and visualization approaches in food safety, which are categorized via the visual analytics pipeline, emphasizing the application of techniques involved in each stage of the pipeline for food safety RAPW. The reviewed literature shows that visual analytics techniques have played an important role and shown great potential in practices of food safety RAPW in recent years. The deep integration of visualization and AI can overcome the limitations of traditional techniques related to RAPW. However, some challenges remain unsolved in this field due to the complexity of food safety risk factors and risk analysis. We also provide the latest ideas for future development of RAPW, such as the visual analysis of multimodal food safety data and the application of AI techniques to the visual analytics pipeline. The objective of this work is to stimulate researchers to propose more excellent visual analytics solutions for more effective food safety RAPW and to provide strong support to food safety regulation.

## Data Availability

Data sharing is not applicable to this article as no datasets were generated or analyzed during the current study.

## Code availability

The code is not applicable. This paper is a review article, and no codes were generated during this work.
